# 295. SARS-CoV-2 Infection in a Referral Cancer Center in Mexico City During the First Year of the COVID-19 Pandemic

**DOI:** 10.1093/ofid/ofab466.497

**Published:** 2021-12-04

**Authors:** Diana Vilar-Compte, Daniel De La Rosa Martinez, Alexandra Martín-Onraet, Carolina Pérez-Jiménez, Beda Islas-Muñoz, Pamela Alatorre-Fernández, Patricia Cornejo-Juarez, Mercedes Aranda-Audelo, Paola Zinser-Peniche, Luis Nuñez-Luna, Erika Ruíz-García

**Affiliations:** 1 Instituto Nacional de Cancerologia, Mexico City, Distrito Federal, Mexico; 2 Instituto Nacional de Cancerología, Mexico City, Distrito Federal, Mexico; 3 Hospital General Dr. Manuel Gea González, Mexico City, Distrito Federal, Mexico

## Abstract

**Background:**

Literature on SARS-CoV-2 infection in cancer patients is scarce in Latin America. This population seems to have a higher risk for adverse outcomes. This study aims to correlate clinical characteristics with outcomes in patients with cancer in a referral center in Mexico.

**Methods:**

We included patients with cancer and confirmed SARS-CoV-2 infection, from April, 19 to December 30, 2020, at the Instituto Nacional de Cancerología, Mexico. Clinical information was obtained from medical and epidemiological records. We conducted a descriptive analysis. For the association between variables with hospitalization, invasive mechanical ventilation (IMV), and mortality; univariate and multivariate logistic regression was performed; odds ratios and 95% confidence intervals were calculated.

**Results:**

Four hundred thirty-three patients were included; 268 (62%) were female, the median age was 55 years. One hundred thirty-five (31%), 130 (30%), and 93 (21%) patients had obesity, hypertension, and diabetes mellitus (DM), respectively. Three hundred forty-one (79%) had solid cancer; 82 (19%) hematological malignancy (HM), and 10 (2%) were under evaluation for cancer diagnosis. One hundred seventy (39%) had advanced or metastatic cancer. One hundred ninety-eight (46%) patients were hospitalized. Risk factors were: age (p= 0.001); woman (p=0.019); HM (p=0.050) and advanced or metastatic cancer (p= 0.041). Fourty-five (10%) patients required IMV. Age (p=0.018); DM (p=0.041); C-Reactive Protein (p= 0.002), and LDH (p= 0.033) were associated with invasive mechanical ventilation. Mortality within 30-days after diagnosis was 19% (82 cases). Associated characteristics were: age (p=0.041); lymphocytes (p=0.049); creatinine (p=0.005) and albumin (p=0.001).

**Conclusion:**

In this study, patients with cancer showed higher mortality, need of hospitalization, and invasive mechanical ventilation compared with groups of patients without cancer. We did not find an increased risk in mortality for hematological malignancies. Although our cohort was younger than others previously reported, age was a strong predictor of adverse outcomes. Variables associated with IMV and death were similar to those previously described in cancer patients with COVID-19.

**Disclosures:**

**All Authors**: No reported disclosures

